# Adaptation and validation of an antibiotic prescribing, peer comparison metric for respiratory tract diagnoses in walk-in clinics: a mixed-methods analysis

**DOI:** 10.1017/ash.2024.436

**Published:** 2024-10-16

**Authors:** Sadie Solomon, Stacey Hockett Sherlock, Gosia Clore, Kimberly C Dukes, Dilek Ince, Kelly M. Percival, Amy M.J. O’Shea, Nathan Shaw, Eli N. Perencevich, Daniel J. Livorsi

**Affiliations:** 1 Department of Internal Medicine, University of Iowa Carver College of Medicine, Iowa City, IA, USA; 2 Center for Access and Delivery Research and Evaluation (CADRE) and the VA Office of Rural Health, Veterans Rural Health Resource Center – Iowa City (VRHRC-IC), Iowa City VA Healthcare System, Iowa City, IA, USA; 3 Department of Pharmaceutical Care, University of Iowa Hospitals and Clinics, Iowa City, IA, USA; 4 Department of Family Medicine, University of Iowa Carver College of Medicine, Iowa City, IA, USA

## Abstract

**Objective:**

Antibiotic overuse is common across walk-in clinics, but it is unclear which stewardship metrics are most effective for audit and feedback. In this study, we assessed the validity of a metric that captures antibiotic prescribing for respiratory tract diagnoses (RTDs).

**Design:**

We performed a mixed-methods study to evaluate an RTD metric, which quantified the frequency at which a provider prescribed antibiotics for RTD visits after excluding visits with complicating factors.

**Setting:**

Seven walk-in clinics across an integrated healthcare system.

**Participants:**

We included clinic visits during 2018–2022. We also conducted 17 semi-structured interviews with 10 unique providers to assess metric acceptability.

**Results:**

There were 331,496 visits; 120,937 (36.5%) met RTD criteria and 44,382 (36.7%) of these received an antibiotic. Factors associated with an increased odds of antibiotic use for RTDs included patient age ≥ 65 (OR = 1.40; 95% CI 1.30–1.51), age 0–17 (1.55, 95% CI 1.50–1.60), and ≥1 comorbidity (OR = 1.22; 95% CI = 1.15–1.29). After stratifying providers by their antibiotic-prescribing frequency for RTDs, patient case-mix was similar across tertiles. However, the highest tertile of prescribers more frequently coded suppurative otitis media and more frequently prescribed antibiotics for antibiotic-nonresponsive conditions (eg, viral infections). There was no correlation between antibiotic prescribing for RTDs and the frequency of return visits (r = 0.01, *P* = 0.96). Interviews with providers demonstrated the acceptability of the metric as an assessment tool.

**Conclusion:**

A provider-level metric that quantifies the frequency of antibiotic prescribing for all RTDs has both construct and face validity. Future studies should assess whether this type of metric is an effective feedback tool.

## Background

Antibiotic overuse is common across the continuum of health care, including in urgent care and other walk-in clinic settings.^1,2^ The overuse of antibiotics drives antibiotic resistance and other antibiotic-related adverse events.^3–5^ Strategies to de-implement antibiotic overuse are therefore needed.

Early efforts at reducing antibiotic overuse in walk-in clinic settings have focused on measuring and reducing antibiotic use for strictly respiratory viral infections.^6^ However, to provide a more comprehensive assessment of antibiotic prescribing, it can be useful to quantify the frequency at which antibiotics are prescribed for all respiratory tract diagnoses (RTDs).^7,8^ The RTD metric quantifies the percentage of patient visits with a RTD that were prescribed an antibiotic. In primary care practices across one university healthcare system, this RTD metric was moderately associated with inappropriate antibiotic prescribing, as defined by manual chart reviews.^8^ Providing feedback on this RTD metric has reduced unnecessary antibiotic use in a stepped-wedge cluster randomized trial in primary care clinics^7^ and an interrupted time series, quasi-experimental study in urgent care settings.^9^ While the findings from these intervention studies are encouraging, further validation of the RTD metric, particularly in different healthcare systems, is necessary before the metric is more widely implemented.

The purpose of this study was to validate the RTD metric among walk-in clinics within our healthcare system. These clinics are almost exclusively staffed by physician assistant-certified (PA-Cs) providers and advanced registered nurse practitioners (ARNPs)—a staffing pattern that differs from the above-mentioned studies.^7–9^

For this study, we focused on assessing the construct and face validity of the RTD metric through qualitative and quantitative analyses. This study was part of a two-year preimplementation assessment to determine whether this new metric should be used to give peer comparison feedback to local walk-in clinic providers.

## Methods

We performed a mixed-methods study, including a quantitative and qualitative analysis, across seven walk-in clinics in the University of Iowa Hospitals and Clinics (UIHC) System. Both aspects of the study were approved by the University of Iowa Institutional Review Board.

### Quantitative analysis

#### Setting and patient population

We performed a retrospective cohort study across seven UIHC walk-in clinics to measure the frequency at which antibiotics were prescribed, specifically for RTDs. Three of these clinics are classified as urgent care (UC) clinics and the remaining four are labeled QuickCare (QC). All clinics have point-of-care testing (eg, rapid tests for influenza and Group A *Streptococcus)* while the three UC clinics also do onsite blood tests and radiographs. Both clinics are primarily staffed by PA-Cs and ARNPs. Conditions treated in these different locations are summarized in Supplemental Table 1 We included in-person patient visits from these clinics for 2018–2022.

#### Overview of antibiotic stewardship initiatives in UC and QC clinics

During 2018–2022, a local antibiotic-prescribing guide was available to all walk-in clinic providers through a website and SmartPhone app. In November 2021, through an initiative unrelated to this study, order sets for common respiratory tract infections were launched and providers started to receive feedback on a “never event” metric that captured antibiotic use for antibiotic-nonresponsive, or tier 3, respiratory conditions.^6^ On a monthly basis, each provider in the walk-in clinics received an e-mail from their supervisor that showed their performance on the never-event metric and other non-stewardship quality metrics. Every quarter providers were e-mailed a weblink that compared their performance on the never-event metric to that of their anonymous peers. Feedback did not label providers as being a “top performer.”

#### Data sources for this study

All patient data was electronically collected from the electronic medical record (Epic Systems Corporation, Verona, WI). Patient comorbidities were identified based on International Classification of Diseases, 10^th^ revision codes (ICD-10) from outpatient and inpatient encounters over the 12 months prior to the index visit using a modified version of the Elixhauser comorbidity index.^10^

#### Visit characteristics

Visits for RTDs were identified by ICD-10 codes (Supplemental Table 2) and mapped to three tiers (tiers 1–3) using a published categorization scheme.^2^ Antibiotics are almost always warranted for tier 1 conditions (eg, pneumonia) and are sometimes warranted for tier 2 conditions (eg, sinusitis, pharyngitis, suppurative otitis media); antibiotics are almost never required for tier 3 conditions (eg, viral infections, asthma). We excluded visits that met the following criteria: (1) Emergency Department (ED) visit or hospitalization ≤ 24 hours after the index walk-in clinic visit; (2) diagnostic code for a non-respiratory infection linked to the index visit; (3) history of leukemia, lymphoma, HIV/AIDS, immunodeficiency, chronic lung disease, hemodialysis, or solid organ/bone marrow transplantation; or (4) any walk-in clinic visit within the prior 31 days.

#### Outcome measures

The primary outcome was the RTD metric, that is, the percentage of patient-visits who received an oral antibiotic agent within 24 hours of a RTD visit. Certain oral antibiotic agents not used for respiratory infections were not captured: fidaxomicin, fosfomycin, metronidazole, nitrofurantoin, rifampin, vancomycin, antifungals, and antivirals. For RTD visits, antibiotic prescribing was sub-analyzed based on specific types of respiratory tract infections, such as non-Group A streptococcal (GAS) pharyngitis, sinusitis, and tier 3 respiratory conditions. Non-GAS pharyngitis and sinusitis were isolated in the analysis due to their high frequency in the cohort and the fact that antibiotics are generally not recommended except in specific circumstances.^11,12^ For provider-level metrics, the primary outcome was aggregated to the level of each unique provider. Secondary outcomes were a) follow-up walk-in clinic visits within 30 days after the index visit and b) a visit to the UIHC ED or inpatient hospital within 30 days after the index visit.

#### Statistical analysis

Patient demographics, provider type, and visit location were summarized by count and percentage among all RTD visits and were stratified by whether an antibiotic was prescribed. We also summarized the frequency of antibiotic prescribing within specific respiratory tract conditions by count and as a percentage of the total. To identify factors associated with antibiotic prescribing for RTDs, we constructed a mixed effects logistic regression model using a logit link with clustering at the patient-level. Alternative model constructions included the use of a provider random effect or nested clustering on patient within provider; however, the model fit for these models, measured by pseudo-likelihood, were inferior to the model reported. Because providers can work in multiple clinic locations, the model did not account for clustering at the clinic level. The model adjusted for antibiotic appropriateness tiers (1–3), as well as other covariates: patient age (categorized as ≤17 years; 18–64 years; and ≥65 years), patient sex (male/female), presence of any comorbidities (yes/no), provider type (ARNP, PA-C, or physician), and clinic type (UC or QC). Visits with an unspecified provider-type were excluded from the model.

Providers with ≥ 100 RTD visits were grouped into tertiles based on their overall antibiotic-prescribing frequency for RTDs. Using the Kruskall-Wallis test, we compared the frequency at which each tertile prescribed antibiotics for (a) all RTD visits, (b) tier 3 RTD visits, (c) sinusitis, (d) non-GAS pharyngitis, and (e) all visits (both RTD and non-RTD). These comparisons were limited to providers who had ≥ 100 qualifying visits for each metric except for sinusitis and non-GAS pharyngitis, which required ≥ 20 qualifying visits for the particular condition per provider. Finally, using Spearman’s correlation, we compared the frequency at which providers prescribed antibiotics for RTDs (limited to providers with ≥ 100 qualifying visits) and the frequency at which their patients had either of the secondary outcomes. All analyses were performed using SAS, version 9.4 (SAS Institute, Cary, NC).

### Qualitative analysis

#### Setting and sample

Our qualitative study design included two rounds of semi-structured, open-ended pre-implementation interviews (approximately 30 minutes in length) with ARNP and PA-C providers at the 7 walk-in clinics.^13^ All ARNP and PA-C providers were invited to participate via presentations given at routine team meetings and by follow-up e-mails. Physicians were not interviewed because they managed a small proportion of all visits and because a physician for these clinics (N.S.) was included on our study team.

The interview guides for both rounds 1 and 2 (supplemental material) were designed to evaluate understanding and perceptions of the usefulness, acceptability, and validity of the RTD metric and graphs showing an anonymized provider’s performance compared with their anonymous peers. Both face validity and construct validity were assessed. Face validity referred to whether the RTD metric was perceived by providers to measure what it claims to measure (ie, the judicious use of antibiotics for RTDs); construct validity referred to whether the RTD metric accurately evaluates the concept of antibiotic stewardship.

Round 2 interviews followed up on responses from round 1 and evaluated preferences for electronic feedback messaging for performance on the RTD metric.

#### Data collection

The qualitative team (S.H.S, K.C.D.) conducted semi-structured, pre-implementation interviews over video-conferencing or telephone from January-March 2023 (round 1) and June–July 2023 (round 2). We conducted 17 interviews: 7 for round 1 and 10 for round 2. Seven providers participated in both interview rounds. Participants were compensated $25 per interview. Interviews were recorded and audio files were transcribed and reviewed for accuracy against the recordings. For each round, interview recruitment was concluded when sufficiency was reached. Sufficiency was determined by evaluating that the response data both answered our research questions and ceased to yield new insights. We solicited feedback from additional providers at routine team meetings. Comments from these meetings were in accordance with interview results.

#### Data analysis

Interview transcripts were imported into MAXQDA 2022 (VERBI Software, Berlin, Germany). We developed a codebook composed of inductive and deductive themes,^14^ and then conducted thematic content analysis using a consensus approach.^15,16^ We reviewed and coded transcripts, then held analysis meetings to review and discuss coding overlap and divergence, in order to apply the codes collectively and systematically to the data using MAXQDA software. Consensus was achieved on all coding.

## Results

### Cohort characteristics and antibiotic-prescribing rates

There were 331,496 in-person visits across 7 walk-in clinics; 120,937 (36.5%) of these were associated with an RTD code and lacked complicating factors (Supplement Figure 1). Antibiotics were prescribed at 96,443 (29.1%) of all visits and 44,382 (36.7%) of RTD visits. The most commonly prescribed antibiotics for RTD visits were amoxicillin (40%) and amoxicillin/potassium clavulanate (27.3%). Characteristics of RTD visits are shown in Table 1.

**Table 1.** Characteristics of respiratory tract diagnoses (RTD) visits at 7 walk-in clinics (2018–2022), stratified by antibiotic prescription status (n = 120,937)

	All RTD visits n = 120,937 (%)	RTD visits with Antibiotic Prescription n = 44,382 (%)	RTD visits without Antibiotic Prescription n = 76,555 (%)
Age group			
0–17	36754 (30.4)	16015 (36.1)	20739 (27.1)
18-64	78681 (65.1)	26524 (59.8)	52157 (68.1)
≥65	5502 (4.5)	1843 (4.2)	3659 (4.8)
Female sex	72336 (59.8)	26241 (59.1)	46095 (60.2)
Race/ethnicity			
White	92,489 (76.5)	35,352 (79.7)	57,137 (74.6)
Black	9,561 (7.9)	2,850 (6.4)	6,711 (8.8)
Hispanic of any race	7,177 (5.9)	2,393 (5.4)	4,784 (6.2)
Asian	4,146 (3.4)	1,074 (2.4)	3,072 (4.0)
Multiracial	3,985 (3.3)	1,484 (3.3)	2,501 (3.3)
Other^a^	3,579 (3.0)	1,229 (2.8)	2,350 (3.1)
Comorbidity^b^	9286 (7.7)	3260 (7.3)	6026 (7.9)
Provider type			
ARNP	81615 (67.5)	31445 (70.9)	50170 (65.5)
PA-C/Physician Associate	34021 (28.1)	10583 (23.8)	23438 (30.6)
Physician	2806 (2.3)	837 (1.9)	1969 (2.6)
Unspecified	2495 (2.1)	1517 (3.4)	978 (1.3)
Visit Location			
QuickCare	90807 (75.1)	34308 (77.3)	56499 (73.8)
Urgent Care	30130 (24.9)	10074 (22.7)	20056 (26.2)
Post-visit encounter type (day 1–30)			
Emergency Department	2488 (2.1)	892 (2)	1596 (2.1)
Hospitalization	625 (0.5)	152 (0.3)	473 (0.6)
Return visit to walk-in clinic	9082 (7.5)	3579 (8.1)	5503 (7.2)

ARNP, advanced registered nurse practitioner; PA-C, physician assistant-certified.

a Other race/ethnicity reflects American Indian, Alaskan Native, Native Hawaiian, Pacific Islander, and unknown.

b Any comorbidity = myocardial infarction, congestive heart failure, peripheral vascular disease, cerebrovascular disease, dementia, rheumatic disease, peptic ulcer disease, mild and severe liver disease, diabetes mellitus (uncomplicated and complicated), hemiparesis, renal disease, renal failure, and nonhematologic malignant cancer.

The overall frequency of antibiotic prescribing by diagnosis tier and for specific types of RTD visits is shown in Table 2. Tier 1 visits were prescribed antibiotics at 95.2% of visits, tier 2 at 50.7% of visits, and tier 3 at 12.8% of visits (Table 2). Supplemental Figure 2 shows how the frequency of antibiotic use changed over time, including after implementation of the never-event metric.

**Table 2.** Respiratory tract condition and frequency at which antibiotics were prescribed across 7 walk-in clinics, 2018–2022 (n = 120,937)

Type of respiratory tract condition	Visits with an antibiotic prescription (n = 44,382)	Total visits (n = 120,937)	% visits with an antibiotic prescription
Tier 1: Pneumonia	**1,277**	**1,342**	**95.2%**
Tier 2	**37,159**	**73,242**	**50.7%**
Suppurative otitis media	15,494	15,848	97.8%
Sinusitis	8,571	9,147	93.7%
Pharyngitis and tonsilitis (+ GAS test)	7,793	7,862	99.1%
Pharyngitis and tonsilitis (GAS test negative or not performed)^a^	5,301	40,385	13.1%
Tier 3	**5,946**	**46,353**	**12.8%**
Bronchitis	1,247	2,799	44.6%
Serous otitis media	1,191	2,993	39.8%
Nasopharyngeal disease, not specified	139	1,121	12.4%
Respiratory symptoms	1,587	14,946	10.6%
Upper respiratory tract infection	1,612	18,356	8.8%
Asthma	116	1,331	8.7%
Rhinitis	12	687	1.8%
Influenza	36	2,832	1.3%
COVID-19	6	1,288	0.5%

GAS, Group A streptococcus.

a A GAS test was not performed in 3,849 visits, which included 729 (18.9%) visits that received an antibiotic prescription. A GAS test had a negative result in 36,536 visits, which included 4,572 (12.5%) visits that received an antibiotic prescription.

### Factors associated with increased antibiotic prescribing at the visit level

At the visit level, patient factors associated with a significantly increased odds of antibiotic use for RTDs included age ≥ 65 vs. age 18–64 (OR = 1.40; 95% CI = 1.30–1.51), age 0–17 versus age 18–64 (OR = 1.55; 95% CI = 1.50–1.60), and having at least one comorbidity (OR = 1.22; 95% CI = 1.15–1.29). Factors protective against an antibiotic prescribing were female sex (OR 0.96; 95% CI 0.93–0.99) and having a QC instead of an UC visit (0.95; 95% CI 0.92–0.98) (Table 3).

**Table 3.** Multivariable analysis of factors associated with antibiotic prescribing for respiratory tract diagnoses visits (n = 118,442)^a^

Factor	Odds ratio (95% CI)	*p*-value
Patient Age (vs. 18–64 years)		
0–17	1.55 (1.50–1.60)	<0.001
65 +	1.40 (1.30–1.51)	
Sex (female v. male)	0.96 (0.93–0.99)	0.0045
Comorbidity^b^ (yes vs. no)	1.22 (1.15–1.29)	<0.001
Provider Type (vs. Physician)		<0.001
ARNP	1.41 (1.27–1.56)	
PA-C	1.04 (0.94–1.14)	
Clinic Type (QC v. UC)	0.95 (0.92–0.98)	0.0038
RTD Tier (vs. Tier 3)		<0.001
Tier 1	164.50 (127.44–212.34)	
Tier 2	7.73 (7.48–8.00)	

ARNP, advanced registered nurse practitioner; PA-C, physician assistant-certified; QC, QuickCare; RTD, respiratory tract diagnosis; UC, Urgent Care.

a Visits where the type of provider was unspecified were excluded from this analysis.

b Any comorbidity = myocardial infarction, congestive heart failure, peripheral vascular disease, cerebrovascular disease, dementia, rheumatic disease, peptic ulcer disease, mild and severe liver disease, diabetes mellitus (uncomplicated and complicated), hemiparesis, renal disease, renal failure, and nonhematologic malignant cancer.

### Comparison of provider antibiotic-prescribing frequency

Providers were stratified into tertiles based on their antibiotic-prescribing frequency for RTDs (Table 4). The mean antibiotic-prescribing frequency was 23.9% (95% CI, 22.5–25.2%) for providers in tertile 1, 33.8% (95% CI, 32.7–35.0%) for providers in tertile 2, and 48.9% (95% CI, 46.3–51.4%) for providers in tertile 3. There were no significant difference in the number of visits, percent of patients with a comorbidity, and patient age distribution across these three groups (Supplemental Table 3).

**Table 4.** Comparison of providers’ antibiotic use and diagnostic coding, after grouping providers into tertiles based on their performance on the **RTD** metric^a^

		Tertile 1 Mean (95% CI) (n = 28 providers)	Tertile 2 Mean (95% CI) (n = 28 providers)	Tertile 3 Mean (95% CI) (n = 28 providers)
Frequency of prescribing antibiotics for specific conditions	**RTD visits, any tier**	23.9 (22.5, 25.2)^**^	33.8 (32.7, 35.0)^**^	48.9 (46.3, 51.4)
	**RTD Tier 3**	5.4 (3.8, 7.0)^**^	10.6 (8.2, 13.0)^**^	18.6 (14.8, 22.3)
	**Sinusitis**	89.5 (84.0, 95.1)^*^	90.8 (86.3, 95.3)^*^	97.8 (96.8, 98.8)
	**Non-GAS pharyngitis**	6.5 (5.3, 7.8)^**^	11.2 (8.8, 13.5)^**^	20.4 (16.8, 23.9)
	**All visits**	21.7 (20.1, 23.2)^**^	27.8 (26.3, 29.3)^**^	35.6 (33.0, 38.2)
Frequency of using specific types of diagnostic codes for RTD visits, %	**Suppurative OM codes**	4.8 (3.6, 6.0)^**^	11.7 (10.0, 13.3)^*^	18.6 (13.6, 23.7)
	**Pneumonia codes**	0.4 (0.3, 0.6)^+^	1.3 (0.6, 2.1)^+^	1.9 (1.0, 2.7)
	**Pharyngitis or sinusitis codes** ^b^	32.6 (27.2, 38.0)^+^	48.5 (45.6, 51.3)^+^	44.2 (39.0, 49.4)
	**Tier 3 RTD codes**	62.2 (56.0, 68.4)^**^	38.5 (35.8, 41.3)^*^	35.3 (30.0, 40.7)

GAS, Group A streptococcus; OM, otitis media; RTD, respiratory tract diagnoses.

a All comparisons include the stated number of providers per tertile, except for sinusitis due to some providers not meeting the requirement of having ≥ 20 visits for sinusitis. For sinusitis, Tertile 1 had 17 providers, Tertile 2 had 23, and Tertile 3 had 25.

b These codes were grouped together because they are part of Tier 2.

+ A comparison to group 3 was only calculated when the p-value was < 0.05 for the overall Kruskall-Wallis test.

* p<0.05 for the comparison between this tertile and Tertile 3.

** p<0.001 for the comparison between this tertile and Tertile 3.

As shown in Table 4, providers in tertile 3 had the highest frequency of coding suppurative otitis media (18.6%), which was significantly more frequent than providers in tertile 1 (4.8%) and tertile 2 (11.7%). Providers in tertile 3 also used tier 3 RTD codes significantly less often than providers in tertiles 1 and 2 (35.3% compared to 62.2% and 38.5%, respectively). Furthermore, providers in tertile 3 had the highest mean frequency of antibiotic prescribing for tier 3 RTD visits, sinusitis, non-GAS pharyngitis, and all visits (both RTD and non-RTD).

### Correlation between antibiotic prescription and subsequent visits

There was no correlation between antibiotic prescribing for RTDs and the frequency of return visits within 30 days (r = –0.01, *P* = 0.96) or a composite outcome of UIHC ED visits and hospitalizations within 30 days (r = 0.01, *P* = 0.95).

### Provider response to RTD antibiotic-prescribing metric

Participants responded with strong consensus that the proposed RTD metric was acceptable. Their responses indicated use of the metric could reinforce more appropriate antibiotic prescribing

- “I guess I would approve of it. It’s capturing the larger picture of just, in general, are we prescribing for things that we might not need to be” (QC PA, #4).
- “I do approve of it. I think it’s a good idea. It kind of keeps us as providers in check and makes sure that we’re not getting a little overzealous with the antibiotics” (UC NP, #8).

Many participants compared the new RTD metric to an existing metric (“never-event metric”) that measures their use of antibiotics for tier 3 respiratory conditions. One provider felt that the new metric may come across as less judgmental: “I think that we’re all a little bit sensitive when you get an email, and then you open it and it says, ‘These are the 15 cases that you did wrong’. This [new metric] is a little more like, ‘Here are some conditions that are generally viral but sometimes require antibiotics’” (UC PA, #1). Others acknowledged receiving feedback on both the never-event metric and the new RTD metric may be synergistic (Table 5).

**Table 5.** Selected quotations from 17 semi-structured interviews with 10 unique providers about the utility, acceptability, and validity of the proposed RTD metric

Theme	Illustrative Quotations
The RTD metric is an acceptable tool for feedback	• “Anytime we can get more data as a group on how we’re doing, it’s gonna be a good thing…I like the long-term view of it” (Urgent Care ARNP, #2).
	• “Yeah. I think that [metric] sounds reasonable. Most of those [respiratory illnesses] are viral, but sometimes antibiotics are needed for some of those conditions” (Urgent Care PA, #1).
	• “Yes, I think it’s a good idea…because we should be treating patients appropriately as best we can for their sake” (QuickCare ARNP, #7).
Importance of data transparency with the RTD metric	• “If a provider got a higher rate (30 percent) and maybe pushes back like, ‘Hey, I was prescribing as I should’, well then I don’t know if there would need to be a deeper dive in some of those chart reviews, like, ‘Well, yes, it does look like this patient had pneumonia. They had a sinus infection for more than 10 days,’ or this or that” (QuickCare PA, #4).
	• “I think the important thing with any feedback is that as the provider, you also have to be able to look at your [data], like having some understanding of the metric” (Urgent Care PA, #9).
Importance of educating providers about the RTD metric before it is used for feedback	• “It’d be nice if that [training about the metric] was in the front-end so then when this feedback comes, it feels like an expectation versus being blindsided. I’ve just listened to people opening emails for the quarterly stuff and it’s just a lot of–it feels very personal. I don’t think that’s how it’s intended to feel, but I don’t think also that people are educated on that this is going to be happening” (Urgent Care PA, #9).
	• “I would think that [demonstration of the metric] might be helpful to those of us that it would be measuring” (QuickCare ARNP, #5).
	• “I think it’s always useful to explain the why and the rationale of why we’re doing something” (Urgent Care ARNP, #3).
Potential synergy between RTD metric and existing never-event metric	• “I’m hoping [the never-event metric] helped decrease some of the over-prescribing of antibiotics because that really did focus in on those times where you really shouldn’t be [prescribing]…I can see both sides being beneficial, for the never events and then this every event” (QuickCare PA, #4).

ARNP, advanced registered nurse practitioner; PA-C, physician assistant-certified.

## Discussion

Our study aimed to validate a metric for our healthcare system that measures the frequency at which walk-in clinic providers prescribe antibiotics for RTDs. We found that providers who performed better on this RTD metric (ie, prescribed antibiotics less frequently for RTDs) also prescribed antibiotics less frequently in general and for respiratory infections that are usually viral in etiology. Better performance on this RTD metric was not associated with more follow-up visits or greater escalation of care. Furthermore, when the RTD metric was presented to providers, it was received positively and was acceptable as a quantitative measure of antibiotic-prescribing practices.

Our study is novel in its attempt to measure perceptions of the RTD metric among providers, which included ARNPs and PA-Cs. Prior research has shown outpatient providers are receptive to receiving antibiotic-prescribing feedback with peer-to-peer comparisons, especially if the feedback is clear, concise, and acknowledges the providers’ good intentions.^17,18^ In our study, providers approved of the RTD metric as an acceptable tool for providing feedback, particularly given its standardized approach to data collection, its decreased vulnerability to coding biases, and its less judgmental approach to evaluation relative to an existing “never event” metric.

We believe our findings indicate the RTD metric has both construct and face validity for evaluating the antibiotic prescribing of walk-in clinic providers. A benefit of the RTD metric is that it allows for more efficient data capture by grouping conditions that necessitate and do not require antibiotics together. This approach to grouping, based on the assumption that the types of RTDs any given provider sees over time will be similar to their peers, may help address provider-level variation in the use of certain diagnostic codes.^19,20^ In addition, our findings can reassure providers that better performance on the RTD metric is not associated with worse outcomes for their patients. Further study is needed to see how the use of this RTD metric for individualized feedback affects prescriber behavior. Based on the findings of at least two studies, it appears that providing feedback on this metric can safely reduce unnecessary antibiotic use.^7,9^

One interesting finding in our analysis was that providers who were in the highest tertile of prescribing frequency for RTDs (tertile 3) were also found to code suppurative otitis media more often than providers in the lower tertiles. While it is possible these providers were, in fact, more frequently seeing this infection type, such a difference between tertiles would be unexpected when the patient case-mix, particularly age, was otherwise similar across the three groups. Because providers in tertile 3 were more likely to prescribe antibiotics for all the conditions we assessed and less likely to use tier 3 codes, their more frequent use of suppurative otitis media codes may reflect a tendency to seek a justification for antibiotics. Inexperience with otoscopy could also explain possible overdiagnosis of suppurative otitis media. Efforts to improve antibiotic prescribing for providers in this tertile should explore how this condition is being diagnosed in their practice.

Our study has several limitations that should be acknowledged. First, we used data from the electronic medical record, which relies on providers entering accurate RTD diagnostic codes. While the metric is designed to capture a broad range of codes, it is possible some providers selected a non-RTD code even though the patient had a respiratory illness. Second, we could not measure all potential factors affecting antibiotic prescribing, including patient symptoms. This is of particular interest for sinusitis, which has prescribing guidelines based on patient presentation.^12^ Third, while the generalizability of these results is potentially impacted by our data set being limited to our specific healthcare network, our findings are in line with previous studies.^21,22^ Fourth, we were unable to capture follow-up visits to outside institutions, so it is possible that patients who did not receive an antibiotic prescription from a UIHC walk-in clinic sought care elsewhere in hopes of receiving a prescription. Finally, our seventeen interviews were limited to 10 providers, which may not fully represent the opinions of all providers in our system. Further study will be needed to see how providers interpret and use this metric.

In conclusion, we adapted a RTD antibiotic-prescribing metric to fit our healthcare system and demonstrated its construct and face validity. This RTD metric effectively captures providers’ prescribing practices and is endorsed by staff as an appropriate tool for evaluating antibiotic use. The ultimate aim of this tool is to reduce unnecessary antibiotic prescribing, and we plan to evaluate its effectiveness for this purpose in a forthcoming clinical trial (ClinicalTrials.gov identifier: NCT06144242).

## Supporting information

Solomon et al. supplementary material Solomon et al. supplementary material

## Data Availability

The data that support the findings of this study are available from the corresponding author, [D.J.L.], upon reasonable request.

## Supplementary material

For supplementary material accompanying this paper visit http://doi.org/10.1017/ash.2024.436. click here to view supplementary material
