# Adaptation and validation of an antibiotic prescribing, peer comparison metric for respiratory tract diagnoses in walk-in clinics: a mixed-methods analysis – ADDENDUM

**DOI:** 10.1017/ash.2024.478

**Published:** 2024-12-26

Sadie Solomon and Stacey Hockett Sherlock are co-first authors for this article.^1^
