# Supplementary material for: Adaptation and validation of an antibiotic prescribing, peer comparison metric for respiratory tract diagnoses in walk-in clinics: a mixed-methods analysis
Source: Antimicrob Steward Healthc Epidemiol. 2024 Oct 16;4(1):e176. doi: 10.1017/ash.2024.436 (PMC11500272; doi:10.1017/ash.2024.436)
Supplement: Solomon et al. supplementary material [file S2732494X24004364sup001.docx]

**Supplemental Material Table of Contents**

| **Description** | **Page number** |
| --- | --- |
| **Supplemental Table 1.** Conditions treated at QuickCare and  Urgent Care Clinics | 2 |
| **Supplemental Table 2**. International Classification of Diseases, Tenth Revision, Clinical Modification (ICD-10-CM) codes for all respiratory tract conditions include in the respiratory tract diagnosis (RTD) metric | 3 |
| **Supplemental Figure 1**. Flowchart of how the study cohort was constructed | 4 |
| **Supplemental Figure 2**. Frequency of antibiotic-prescribing frequency for visits associated with respiratory tract diagnoses across 7 walk-in clinics, 2018-2022 | 5 |
| **Supplemental Table 3.** Provider-level volume and types of patients seen by providers, after grouping providers into tertiles based on their performance on the respiratory tract diagnoses (RTD) metric | 6 |
| **Interview Guide, Round #1** | 7-9 |
| **Interview Guide, Round #2** | 10-12 |
| **Sample feedback graphs shown during interviews** | 13-18 |

**Supplemental Table 1. Conditions treated at QuickCare and Urgent Care Clinics**

| **QuickCare clinics** | **Urgent Care Clinics** |
| --- | --- |
| Head, ear, and eye conditions   - - Ear infections   - Earaches   - Ear wax buildup   - Eye abrasions   - Eye infections   - Pink eye   - Styes - Minor injuries   - Below-the-neck laceration repair   - Minor burns   - Minor musculoskeletal injuries   - Sprains   - Fracture - Stomach and genital urinary conditions   - Bladder infection   - Constipation   - Diarrhea, nausea and vomiting   - Stomach flu   - Sexually transmitted disease (STD)   - Urinary burning   - Frequent urination - Skin conditions   - Abscess   - Athlete’s foot   - Cold sores   - Insect bites   - Rashes   - Itchy skin   - Ringworm   - Skin infection   - Sunburn - Respiratory illness   - Flu symptoms   - Bronchitis   - Chest/nasal congestion   - Common cold   - Cough   - Seasonal allergies   - Sinus infections   - Sore throat/laryngitis   - Pneumonia   - Respiratory syncytial virus (RSV) | All the conditions listed under QuickCare clinics plus:   - Performing x-rays - Treating broken bones - Treating strains and sprains - Assisting with dehydration (intravenous fluids) - Treating cuts and scrapes (Stitches) |

**Supplemental Table 2. International Classification of Diseases, Tenth Revision, Clinical Modification (ICD-10-CM) codes for all respiratory tract conditions include in the respiratory tract diagnosis (RTD) metric**

|  | **ICD-10 Code** |
| --- | --- |
| **Tier 1 diagnoses: antibiotics are almost always indicated** | A37 (whooping cough), A48.1 (Legionnaires’ disease), B0.12, B0.52, B0.681 (viral pneumonia), H70 (mastoiditis), J05, J05.1 (acute epiglottitis), J09.x1, J10.0, J11.0, J12, (viral pneumonia), J13-J18 (bacterial pneumonia), J36 (peritonsillar abscess), J39.0/J39.1 (pharyngeal abscess), J47.0 and J47.1 (bronchiectasis with infection/exacerbation) |
| **Tier 2 diagnoses: antibiotics may be indicated** | H66, H67, H73.0, H73,1, H73.2 (suppurative/unspecified otitis media), J01 (acute sinusitis), J02 and J03 (pharyngitis and tonsillitis), J03.8/J03.9 (acute tonsillitis), J32 (chronic sinusitis), J44.0/J44.1 (chronic obstructive pulmonary disease with acute exacerbation) |
| **Tier 3 diagnoses: antibiotics are almost never indicated** | B0.53, H65, H68 and H69 (non-suppurative otitis media), H92 (ear symptoms), J00 (acute nasopharyngitis), J04 (acute laryngitis/tracheitis), J05.0 (acute obstructive laryngitis), J06 (acute upper respiratory infections), J09/J10/J11 except as noted in tier 1 (influenza infection), J20 (acute bronchitis), J21 (acute bronchiolitis), J22 (other acute lower respiratory infection), J30 (vasomotor/allergic rhinitis), J31 (chronic rhinitis/nasopharyngitis/pharyngitis), J34.89, J34.9 (other nasal disorders), J35 (chronic diseases of tonsils/adenoids), J37 (chronic laryngitis/tracheitis), J39.2 (other upper respiratory tract diseases), J40 (unspecified bronchitis), J41/J42 (chronic bronchitis), J43 (emphysema), J44.89-J44.9 (chronic obstructive pulmonary disease ), J45 (asthma), J47.9 (bronchiectasis), J98.01, J.98.8, J98.9, J99 (other respiratory disorders), R04-R06.2, R06.82, R06.89, R06.9, R07, R07.0, R07.1, R07.81, R09.02, R09.1, R09.3, R09.8 (respiratory symptoms), U07.1, U09.9, Z20.822 (COVID-19), Z87.09 (history of respiratory disease) |

**Supplemental Figure 1. Flowchart of how the study cohort was constructed**

All in-person, walk-in clinic visits during 2018-2022

(excluding patients seen in the Emergency Department or hospitalized within 24 hours)

n=331,496

*Chronic lung disease was defined by ICD-10 codes for chronic sinusitis, chronic bronchitis, emphysema, chronic obstructive pulmonary disease, bronchiectasis, and different types of hypersensitivity pneumonitis.

**Exclude**

**Exclude**

Final cohort of respiratory tract diagnosis visits (n=120,937)

Visits with a non-respiratory diagnosis (n=192,909)

Visit for a respiratory tract diagnosis within the prior month (n=10,376)

Visits with a non-specified provider (n=110)

Comorbidities (n=7164) *below categories are not mutually exclusive

- Leukemia or lymphoma (n=521)
- HIV/AIDS or immunodeficiency (n=694)
- Chronic lung disease (n=5,963)*
- Hemodialysis (n=155)
- Solid organ or bone marrow transplant (n=276)

Visits with a respiratory tract diagnosis (n=138,587)

**Supplemental Figure 2. Frequency of antibiotic-prescribing for visits associated with respiratory tract diagnoses across 7 walk-in clinics, 2018-2022**

**Frequency of prescribing antibiotics**

**Never-event metric introduced**

**Supplemental Table 2. Provider-level volume and types of patients seen by providers, after grouping providers into tertiles based on their performance on the respiratory tract diagnoses (RTD) metric**

|  | **Tertile 1** | **Tertile 2** | **Tertile 3** | **p-value**^1^ |
| --- | --- | --- | --- | --- |
| **Number of providers in tertile** | 28 | 28 | 28 | N/A |
| **Antibiotic-prescribing frequency for RTD visits, provider-level mean (95% CI)** | 22.5%  (21.2-23.8) | 30.3%  (29.3-31.2) | 37.8%  (36.6-39.1) | N/A |
| **Number of visits per provider, mean (95% CI)** | 1241  (750-1732) | 1595  (1069-2122) | 1384  (953-1814) | 0.34 |
| **Types of patients, provider-level**  Any comorbidity, mean % (95% CI)^2^  Age ≤ 17, mean % (95% CI)  Age 18-64, mean % (95% CI)  Age ≥ 65, mean % (95% CI) | 7.8% (7.1-8.5)  31.2% (29.7-32.7)  64.0% (62.1-65.8)  4.8% (4.0-5.6) | 7.6% (6.9-8.4)  31.1% (28.6-33.7)  64.2% (61.4-67.0)  4.7% (4.1-5.3) | 8.7% (7.6-9.9)  31.9% (29.9-33.9)  63.0% (61.0-64.9)  5.1% (4.6-5.6) | 0.37  0.53  0.61  0.21 |

Abbreviations: CI confidence interval; RTD respiratory tract diagnoses

1. The Kruskall-Wallis test was used to make comparisons across the three tertiles.
2. Any comorbidity = myocardial infarction, congestive heart failure, peripheral vascular disease, cerebrovascular disease, dementia, rheumatic disease, peptic ulcer disease, mild and severe liver disease, diabetes mellitus (uncomplicated and complicated), hemiparesis, renal disease, renal failure, non-hematologic malignant cancer

**Interview Guide, Round #1**

***Qualitative Recruitment***

*These interviews will be conducted with Urgent Care and QuickCare clinicians. Approximately 15 interviews will be conducted. Interviews will last between 15-30 minutes depending on provider availability.* *Interview participants will be given $25 giftcards.*

**Intro script:**
This is considered a research project. Your name will not be included with any information, and you are free not to answer any questions. I will ask if I can record the interview, and you are free to say no.

Do you have any questions for me before we begin?
Do I have your permission to record? *start audio recording device*

*SECTION A: Demographic questions*

1. What is your primary role at [name of facility]?
2. Do you work at more than one facility? Which ones?
3. How long have you worked at your current facility? How long in this role?

******

*SECTION B: Questions about Metric report*

[INTERVIEWER PULLS UP THE METRIC REPORT]

1. What are your initial reactions to this graph?
2. [Comprehension Tasks]
   1. What level of performance does this figure show?
3. [Thoughts about goal/target/metric]
   1. What do you think about the goal/target?
   2. What do you think about the performance metric?
4. [Interpretation of Comparisons]
   1. Graph 1: This figure shows the frequency at which a provider prescribed antibiotics for respiratory conditions over the past few years in comparison to the average prescribing rate for the entire [urgent care or Quick Care] clinic.
      1. Is this a meaningful comparison?
      2. If not, what would be meaningful?
   2. Graph 2: This figure shows a provider’s prescribing data for the past 2 months in comparison to similar data from their peers. The table shows how respiratory illness visits were coded and which codes were associated with antibiotic prescriptions.
      1. Is this a meaningful comparison?
      2. If not, what would be meaningful?
   3. How do you feel about receiving feedback on your antibiotic-prescribing and being compared to your peers?
   4. [Design quality] Does this sample feedback report make sense to you? Do you feel it is accurate? What kind of improvements or changes would you suggest?
5. [Time Interval]
   1. What is the optimal reporting interval for this feedback? Every two months is probably the target. Would you prefer to see feedback more or less often [Monthly, Quarterly]?
   2. What range of dates would you like to see? [1 year, more, less?]
6. [Language and Framing]
   1. How would you describe the tone of the messaging of this report? [Positive, Negative, Neutral?]
   2. Do you feel these types of reports might influence your future prescribing decisions for acute respiratory infections (ARIs)? Why or why not?
7. [Delivery/Viewing]
   1. How would you like to receive these reports? Options include receiving a static report as an attachment to an e-mail, or receiving an email with a weblink to a dashboard. Which of these options would you prefer?
   2. If this report were to be implemented as part of routine practice in your setting, would you have time to open an e-mail from our team and review your feedback report? How often would you review a report like this?
   3. What would encourage you to read these reports regularly?
8. Is there anything else you'd like to add?

*****

*SECTION C: Questions about prescribing decision factors*

1. **Available resources:** Do you feel like you have the diagnostic tools at hand to distinguish respiratory infections that are viral versus bacterial? [Prompt: testing for Group A strep, rapid testing for viruses, chest x-ray]
2. **Self-efficacy:** Do you feel you have access to guidelines about how to prescribe antibiotics appropriately? What kinds of guidelines do you use?
3. **Opinion leaders**: Is there anyone in this clinic who influences how you or others think about your antibiotic-prescribing (such as a champion)? If so, who? Please explain. Do you consult with other providers or leadership to make prescribing decisions?

**Optional questions, as time allows**

1. How do you decide to prescribe antibiotics for sinusitis? For pharyngitis? For otitis media?
2. How do you decide whether to prescribe antibiotics in patients with an acute cough illness?
3. What are barriers to determining if antibiotics are indicated or not for acute respiratory infections (ARIs)?
4. What are some of the reasons that antibiotics are prescribed for acute respiratory infections (ARIs) when antibiotics are not indicated or indication is unclear? (such as patient preferences)
5. Some providers have diagnostic codes they prefer to use. How do you decide which diagnostic code to use?

*****
**End of interview:**
*Who else at your facility should we talk with?*
*Thank you for your time today.*

**Interview Guide, Round #2**

***Qualitative Recruitment***

*These interviews will be conducted with Urgent Care and QuickCare clinicians. Approximately 15 interviews will be conducted. Interviews will last between 15-30 minutes depending on provider availability.* *Interview participants will be given $25 giftcards.*

**Intro script:**
This is considered a research project. Your name will not be included with any information, and you are free not to answer any questions. I will ask if I can record the interview, and you are free to say no.

Do you have any questions for me before we begin?
Do I have your permission to record? *start audio recording device*

*SECTION A: Demographic questions*

1. What is your primary role at [name of facility]?
2. Do you work at more than one facility? Which ones?
3. How long have you worked at your current facility? How long in this role?

******

*SECTION B: Questions about Commitment letter, Patient education poster, Patient education handout, Email messaging*

In the first round of interviews, clinicians identified some ways the clinic could help support them in antibiotic prescribing. We'd like to show you drafts of some resources and ask what you think about them.

[INTERVIEWER SHOWS THE DOCUMENT/RESOURCE]

1. This commitment letter is meant to be visible to both patients and providers. What are your initial reactions to this letter?

a. Do you feel it would be useful to have as a resource?

b. How do you think patients would respond to the letter?

c. Who should sign the letter? Would you be willing to sign it?

d. Where in your clinic should the letter be displayed?

1. What are your initial reactions to this poster?

a. Do you feel it would be useful to have as a resource?

b. How do you think patients would respond to the poster?

1. What are your initial reactions to this patient education handout?

a. Do you feel it would be useful to have as a resource?

b. How do you think patients would respond to the poster?

1. [Response to options for email messaging; show each message option individually]
   1. These messages show a provider’s prescribing data for the past 2 months in comparison to similar data from their peers. One of these messages comes from prior research studies while the other one is new.
2. [Language and Framing, for each email messaging option]
   1. How would you describe the tone of the messaging of this email? [Positive, Negative, Neutral?]
   2. Do you feel these types of messages might influence your future prescribing decisions for acute respiratory infections (ARIs)? Why or why not?
   3. Is this a meaningful comparison? If not, what would be meaningful?
   4. How do you feel about receiving feedback on your antibiotic-prescribing and being compared to your peers?
   5. [Design quality] What kind of improvements or changes would you suggest?
3. [Delivery/Viewing for email]
   1. If this email were to be implemented as part of routine practice in your setting, would you have time to open an email from our team and review your feedback report? How often would you review a report like this?
   2. What would encourage you to read these emails regularly?
4. To help explain our new metric, we plan to offer some education about the rationale for the metric and how it is measured. What are your thoughts about that? (Should this be presented on a clinic call, other options? Would education be useful, not necessary? Would education about the metric influence acceptance of its validity?)
5. Some providers have trouble explaining to a patient why he/she will not prescribe an antibiotic when the patient believes an antibiotic is needed. These providers may be able to learn from other providers who are more comfortable handling these types of situations. What are your thoughts about having a local discussion among providers about how to effectively communicate with patients when antibiotics are not indicated? (Would this be useful, not necessary? Who should lead these discussions?)
6. What are some barriers to optimal antibiotic-prescribing for ARIs? What are some facilitators to optimal antibiotic-prescribing for ARIs?
7. Is there anything else you'd like to add?

*****

*SECTION C: Optional questions, as time allows*

1. **Available resources:** Do you feel like you have the diagnostic tools at hand to distinguish respiratory infections that are viral versus bacterial? [Prompt: testing for Group A strep, rapid testing for viruses, chest x-ray]
2. **Self-efficacy:** Do you feel you have access to guidelines about how to prescribe antibiotics appropriately? What kinds of guidelines do you use?
3. **Opinion leaders**: Is there anyone in this clinic who influences how you or others think about your antibiotic-prescribing (such as a champion)? If so, who? Please explain. Do you consult with other providers or leadership to make prescribing decisions?
4. How do you decide to prescribe antibiotics for sinusitis? For pharyngitis? For otitis media?
5. How do you decide whether to prescribe antibiotics in patients with an acute cough illness?
6. What are barriers to determining if antibiotics are indicated or not for acute respiratory infections (ARIs)?
7. What are some of the reasons that antibiotics are prescribed for acute respiratory infections (ARIs) when antibiotics are not indicated or indication is unclear? (such as patient preferences)
8. Some providers have diagnostic codes they prefer to use. How do you decide which diagnostic code to use?

*****
**End of interview:**
*Who else at your facility should we talk with?*
*Thank you for your time today.*
